# Examining the Differences in Frequency of Whole Fruit or 100% Fruit Juice Intake among United States Adults With and Without Cardiovascular Disease Risk Factors: Analysis of 2019 Behavioral Risk Factor Surveillance System Data

**DOI:** 10.1016/j.cdnut.2026.109517

**Published:** 2026-08-19

**Authors:** Brendan J Kesler, Julie M Hess, Anne Bodensteiner, Daniel G Palmer, Desiree Tande

**Affiliations:** 1 Department of Nutrition and Dietetics, University of North Dakota, Grand Forks, ND, United States; 2 United States Department of Agriculture, Agriculture Research Service, Grand Forks Human Nutrition Research Center, Grand Forks, ND, United States; 3 University of North Dakota, Grand Forks, ND, United States

**Keywords:** fruits and vegetables, cardiovascular disease, coronary heart disease, fruit, fruit juice

## Abstract

**Background:**

Nearly 80% of Americans do not meet recommendations for fruit, an important source of fiber, potassium, and vitamin C. In the 2020–2025 Dietary Guidelines for Americans, the fruit group included whole fruit (fresh, frozen, dried, and canned) as well as 100% fruit juice (“juice”). Approximately half of reported fruit intake comes from whole fruit (“fruit”), whereas the other half comes from juice, sugar-sweetened beverages, and diet beverages.

**Objectives:**

The objective of this study was to investigate whether frequency of fruit or juice intake differs among adults with and without cardiovascular disease (CVD) or CVD risk factors.

**Methods:**

Data from 371,038 United States adults from the 2019 Behavioral Risk Factor Surveillance System were used to identify consumers of fruit and juice, frequency of fruit or juice intake, and those with high blood pressure, elevated blood cholesterol, angina/coronary heart disease (CHD), heart attack, or stroke. Independent sample *t* tests and linear regression were used to determine whether mean fruit consumption or juice consumption frequency differed between groups with and without CVD or CVD risk factors.

**Results:**

Fruit consumption frequency was lower in adults who self-reported high blood pressure (*P <* 0.0001), high cholesterol (*P <* 0.0001), heart attack (*P <* 0.0001), CHD (*P* = 0.0020), or stroke (*P <* 0.0001), before and after controlling for age, education, income, and smoking. Juice consumption frequency was higher in adults who self-reported high blood pressure (*P <* 0.0001), heart attack (*P <* 0.0001), CHD (*P =* 0.0166), or stroke (*P <* 0.0001). After controlling for sociodemographic characteristics, significant differences in juice intake frequency remained only for high cholesterol and heart attack.

**Conclusions:**

Adults with less favorable CVD status reported lower fruit intake frequency and, for some indicators, differences in juice intake frequency. Additional research is warranted to examine how CVD status affects fruit intake frequency, pinpoint potential mechanisms, and identify strategies to increase fruit consumption in the United States population.

## Introduction

Fruit is a component of recommended dietary patterns throughout the world, and its contributions to health promotion and disease prevention are well established. In the United States, the 2020–2025 Dietary Guidelines for Americans (DGA) recommended 2 cup-equivalents of fruit per day for Americans eating a 2000 kcal healthy United States-style dietary pattern [1]. The 2025–2030 DGA echoes this recommendation [2]. Yet ∼80% of the United States population aged ≥2 y does not meet fruit recommendations and consumes <1 cup-equivalent of fruit per day [3]. More Americans meet fruit intake recommendations in early life (ages 1–8 y), but fruit intake declines to <1 cup-equivalent per day during elementary school years where it remains through adulthood [3,4]. Only 12.3% of United States adults meet fruit intake recommendations with a mean consumption frequency of only once per day [5]. Fruit intake in the United States population is comprised of approximately one-half whole fruit with the other half of fruit intake coming from 100% fruit juice and sugar-sweetened and diet beverages [3].

The definition for what constitutes a fruit serving varies throughout the world, and the inclusion or exclusion of juice is a key point of differentiation among global fruit intake guidelines. The 2020–2025 DGA defined a serving of fruit to be 1 cup-equivalent of raw fruit, 1 cup-equivalent of 100% fruit juice (“juice”), or one-half cup-equivalent of dried fruit [1]. Although juice is part of the fruit category, the recommendations also state that fruit should be consumed in whole form as often as possible and, at a minimum, “at least half of the recommended amount of fruit should come from whole fruit, rather than 100% juice,” implicitly allowing for half of the fruit servings to come from juice [1]. The 2025–2030 DGA includes whole fruit (fresh, frozen, canned, and dried) and fruit juice in the description of the fruit group but lists only serving examples for raw and dried fruit and explicitly recommends limiting and diluting juice [2].

Other countries vary in their juice recommendations due, in part, to the high sugar content of juice [6,7]. The WHO recommends <5% of total energy come from foods containing free sugars, which includes juice, to achieve the most health benefits [8]. Countries including the United Kingdom, Ireland, Spain, France, Hungary, Austria, and Denmark recommend that a maximum of 1 serving of juice contribute to daily fruit servings [6]. In the Netherlands, juice is included in the sugar-containing beverage category rather than the fruit category because of its sugar content [9].

Fruit is also a leading source of dietary fiber for Americans aged ≥2 y [3]. The fruit group contributes 10.5% of dietary fiber intake for females ≥2 y and 9.3% of dietary fiber intake for males ≥2 y, based on NHANES 2011–March 2020 data [10]. Fiber has been a nutrient of public health concern for Americans because of low intake since the 2005 DGAs [1,[11], [12], [13]]. Over 80% of female adults and 90% of male adults do not meet the adequate intake for dietary fiber [3]. During the juicing process, most dietary fiber is removed from fruit, which creates a compositional difference between fruit and juice [6]. As an example, the USDA National Nutrient Database for Standard Reference Legacy database shows that 1 cup of 100% orange juice contains 0.5 g of fiber, whereas 1 cup of orange segments contains 3.6 g of fiber [14]. Because of the significant underconsumption of dietary fiber in the United States and dietary fiber’s well-established link to positive health outcomes, including reducing risk of coronary heart disease (CHD), the removal of fiber from fruit is an important dietary consideration for public health [1].

In addition to dietary fiber, another important nutritional attribute of fruit may be its associated phytochemicals, such as polyphenols. Polyphenols are the most abundant form of antioxidant available in the diet, comprising an estimated 93% of total antioxidant intake [15]. Fruit is the largest dietary source of polyphenols containing as much as 200 to 300 mg of polyphenols per 100 g of fruit [16]. However, the types of polyphenols can differ by fruit form as a whole fruit contains predominantly nonextractable polyphenols, whereas juice contains primarily extractable polyphenols [16,17]. These unique forms of polyphenols have different physiological behaviors with extractable polyphenols being more bioavailable in the small intestine and nonextractable polyphenols, which make up the largest portion of total available polyphenols [15], being resistant to digestion in the stomach and small intestine and reaching the colon primarily intact [18,19]. Much of the research focused on polyphenols evaluates only the content and impact of polyphenols that can be extracted using aqueous-organic solvents (e.g., extractable polyphenols) [15]. Nonextractable polyphenols are found in those extraction residues. Within the bacterial environment of the colon, nonextractable polyphenols can be fermented by microbiota, modulating abundance and species of intestinal microbiota and can produce beneficial metabolites [7,18]. The different primary polyphenol types in whole fruit and juice highlight the potential importance of whole fruit as a source of not only dietary fiber but also the nonextractable polyphenols that are bound to dietary fiber.

The fiber, bioactive compounds, and nutrients found in whole fruit work synergistically to promote health [20]. In addition to being a key dietary source of nutrients, fruit intake mitigates risk of multiple chronic diseases, including cardiovascular disease (CVD) [[20], [21], [22]]. The role of whole fruits in the prevention of CVD is of critical importance as CVD is the leading cause of death globally [23]. Although the association between whole fruit consumption and reduced risk of CVD is well established [24], the impact of juice intake compared with whole fruit intake on CVD risk has not been fully elucidated [7]. Conflicting results indicate that further research is required [7,25]. In addition, how diets, specifically fruit and juice intake, differ among individuals with and without CVD has also not been fully investigated.

The aim of this study was to increase epidemiological understanding of potential relationships between CVD and fruit intake frequency by type (“whole fruit” compared with “juice”). Our research examined whole fruit consumption frequency, juice consumption frequency, and CVD risk factors and measures in United States adults using 2019 Behavioral Risk Factor Surveillance System (BRFSS) data. We hypothesized that whole fruit consumption frequency will be lower in adults with less favorable cardiovascular measures and that juice consumption frequency will be higher in adults with less favorable cardiovascular measures.

## Methods

BRFSS is a series of telephone surveys collecting information on health-related chronic conditions, risk behaviors, access to care, and use of preventive services from the noninstitutionalized adult population in the United States [26]. This cross-sectional study was conducted according to the guidelines laid down in the Declaration of Helsinki and all procedures involving research study participants were approved by the University of North Dakota Institutional Review Board (IRB0005041).

During 2019, the BRFSS collected data from 418,268 adults, aged ≥18 y, across all 50 states as well as Guam, Puerto Rico, and District of Columbia [26]. The only known limitation of the 2019 data is that the state of New Jersey was unable to collect the minimum data requirement for inclusion and is therefore not included in the aggregate data set. The 2019 BRFSS data were selected for analysis, because at initiation of this project, it was the latest survey that included the following modules relevant to the research: the Hypertension Awareness Module, Core Section 4; the Cholesterol Awareness Module, Core Section 5; and the Fruits and Vegetables Module, Core Section 12. These modules, which are not included in all BRFSS annual surveys, were critical to address our research question.

A cross-sectional, secondary data analysis was performed using self-reported data from the 2019 BRFSS to explore if the consumption frequency of whole fruit and juice was different between groups with and without CVD or groups with and without risk factors for CVD. Table 1 shows the BRFSS modules, variables, questions, and responses used in the analysis to determine if participants self-reported the presence of a CVD risk factor or measure and to determine frequency of whole fruit and juice consumption.

**TABLE 1:** 2019 Behavioral Risk Factor Surveillance System Modules and survey questions used for analysis of associations among frequency of whole fruit and 100% fruit juice consumption and cardiovascular health indicators

Module	Section and question numbers	Variable name	Question text	Responses used
Hypertension awareness	Core Section 4: C04.01	BPHIGH4	Have you ever been told by a doctor, nurse, or other health professional that you have high blood pressure?	1 (Yes), 3 (No)
Cholesterol awareness	Core Section 5: C05.02	TOLDHI2	Have you ever been told by a doctor, nurse, or other health professional that your blood cholesterol is high?	1 (Yes), 2 (No)
Chronic health conditions	Core Section 6: C06.01	CVDINFR4	Has a doctor, nurse, or other health professional ever told you that you had a heart attack also called a myocardial infarction?	1 (Yes), 2 (No)
Chronic health conditions	Core Section 6: C06.02	CVDCRHD4	Has a doctor, nurse, or other health professional ever told you that you had angina or coronary heart disease?	1 (Yes), 2 (No)
Chronic health conditions	Core Section 6: C06.03	CVDSTRK3	Has a doctor, nurse, or other health professional ever told you that you had a stroke?	1 (Yes), 2 (No)
Fruits and vegetables	Core Section 12: C12.01	FRUIT2	Not including juices, how often did you eat fruit?	Quantitative responses
Fruits and vegetables	Core Section 12 C12.02	FRUITJU2	Not including fruit-flavored drinks or fruit juices with added sugar, how often did you drink 100% fruit juice such as apple or orange juice?	Quantitative responses

The following survey responses were not subpopulations of interest in the analysis for variables BPHIGH4, TOLDHI2, CVDINFR4, CVDCRHD4, and CVDSTRK3: Don’t Know/Not Sure (all variables), Refused (all variables), Not Asked or Missing (all variables), Yes, but female told only during pregnancy (BPHIGH4 only), Told borderline high or prehypertensive (BPHIGH4 only). Additionally, note that Yes and No groups are based on self-response and self-report of diagnosis of disease or risk factors. For the variables FRUIT2 and FRUITJU2, the raw data response options were: 1 (self-reported per day amount), 2 (self-reported per week amount), 3 (self-reported per month amount), 300 (Less than once a month), 555 (Never), 777 (Don’t Know), and 999 (Refused). These are the original data collected; analysis variables were calculated to a frequency/day amount in the calculated variables provided with the data set.

Utilizing the questions shown in Table 1 from the Hypertension Awareness Module, Cholesterol Awareness Module, and Chronic Health Conditions Module, groups were created for comparison by utilizing a survey response of “yes” for group 1, indicating the presence of a CVD risk factor or marker, and utilizing a response of “no” for group 2, indicating the absence of a CVD risk factor or marker. The Demographics Module of the BRFSS was also used to describe population demographics and remove any survey participants who reported being pregnant. The measures for CVD risk factors included in this study were high blood pressure and high cholesterol. The CVD disease measures included in this study were myocardial infarction (i.e., heart attack), angina or CHD, and stroke.

### Statistical analysis

Statistical analyses were performed utilizing SAS 9.4 software (Copyright 2023 SAS Institute Inc). BRFSS data (combined Land Line and Cell Phone data) were downloaded in SAS transport form directly into SAS. The calculated variables for whole fruit consumption frequency (FRUTDA2_) and juice consumption frequency (FTJUDA2_), which standardize survey participant responses into a calculated per-day frequency, were truncated to remove the 2 decimal places. To do so, the original values were divided by 100 to remove the 2 implied decimal places.

A filter was then applied to remove any survey participants missing data on fruit consumption frequency or who met the Centers for Disease Control’s (CDC) recommended exclusion criteria of having a reported frequency of fruit consumption >16 fruits per day (_FRUITE1). Utilizing the demographics variable (PREGNANT), any survey participants who reported being pregnant were also removed from the dataset. After each data transformation and applied filter, frequencies were calculated and checked against the CDC codebook to ensure data integrity. Frequencies for age group, race/ethnic group, sex, education group, income group, and CVD risk factors/measures were calculated using PROC SURVEYFREQ.

Finally, indicator variables corresponding to the “yes” and “no” categories for each CVD risk factor/measure were created to make inference on these subpopulations while incorporating information from the other categories for the SEs. The race/ethnicity variable (_RACE) was recoded as White – non-Hispanic, Black – non-Hispanic, Hispanic, other race – non-Hispanic, multiracial – non-Hispanic, and don’t know/not sure/refused/missing.

Independent samples *t* tests were performed on each cardiovascular condition or CVD risk factor using PROC SURVEYREG. The *t* test determined whether the mean whole fruit consumption frequency or mean juice consumption frequency significantly differed between 2 groups, one with and the other without CVD or CVD risk factors.

Regression models including independent variables of CVD group (“Yes” or “No”), age group, race/ethnic group, education group, income group, and smoking status were used to predict (whole) fruit (juice) consumption as a sensitivity analysis using PROC SURVEYREG. Contrasts were used to calculate differences in estimated marginal means between the CVD groups while accounting for these additional covariates.

The strata (_STSTR), cluster (_PSU), and weight (_LLCPWT) variables were used to account for the complex survey design. *P* values were adjusted for the 5 CVD variables with Bonferroni corrections. A significance level of *α = 0.05* was used.

## Results

**FIGURE 1:**
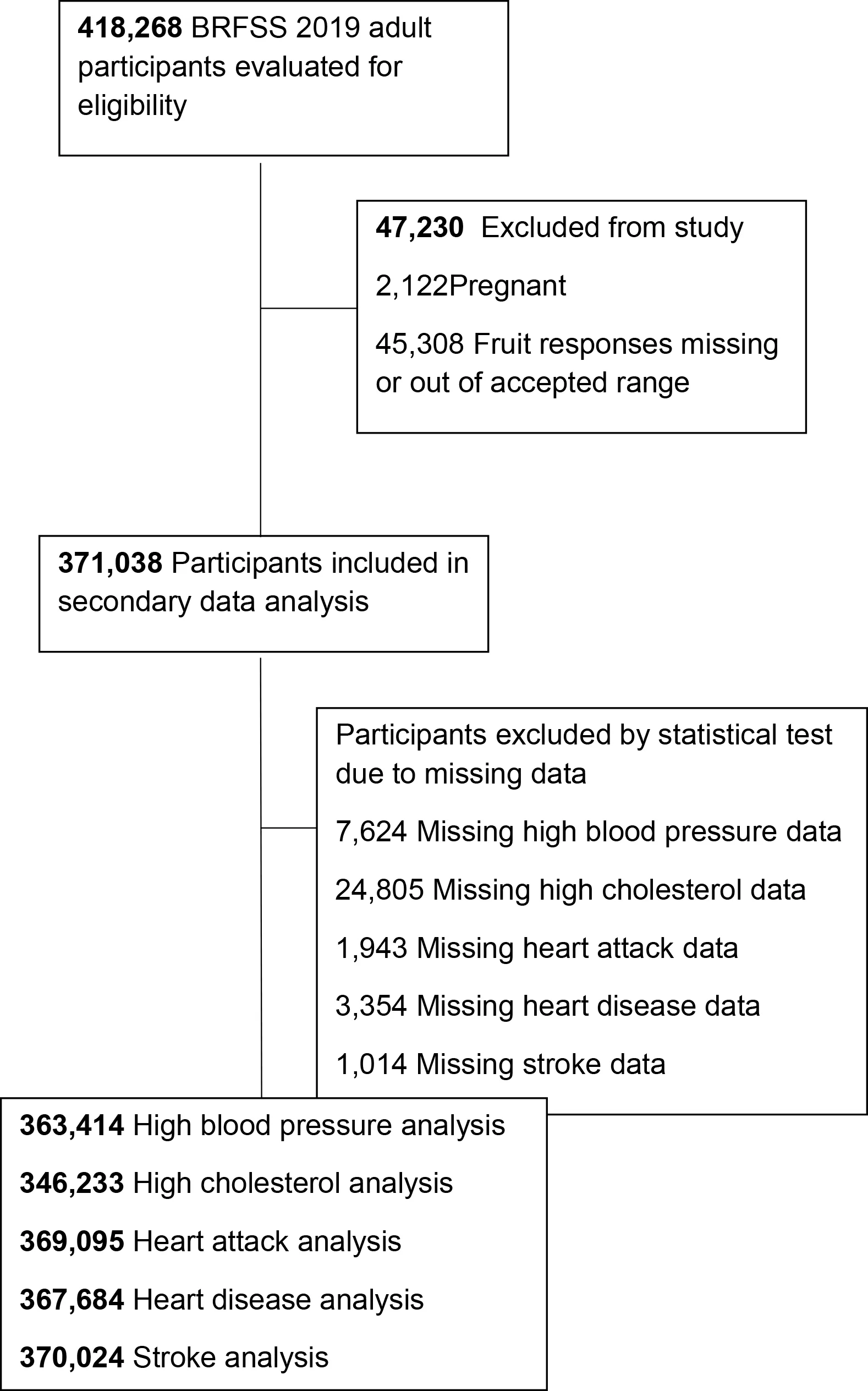
Flowchart of the 2019 Behavioral Risk Factor Surveillance System study participants included in analysis of the prevalence of cardiovascular disease, cardiovascular disease risk factors, and intake of whole fruit and fruit juice.

After the removal of outliers, the total number of BRFSS participants included in the analysis was 371,038 of which 49.0% were male (Figure 1). Table 2 displays the categorized demographics for the population including age, race, education, and income. The majority (21.8%) of participants fell into the ≥65 y age range, were White – non-Hispanic (62.3%), had attended college or technical school (31.2%), and reported an annual income of ≥$50,000 (44.1%) (as shown in Table 2).

**TABLE 2:** Characteristics of 2019 BRFSS survey participants included in analysis (*N* = 371,038)

Characteristic	Frequency (*n*)	Weighted frequency (*n*_*w*_)	Percent of total
Age range (y)			
18–24	21,699	26,437,493	12.1
25–34	37,433	36,789,788	16.8
35–44	43,332	35,221,840	16.1
45–54	54,116	35,257,588	16.1
55–64	75,894	37,060,888	17.0
≥65	138,564	47,699,547	21.8
Race			
White—non-Hispanic	279,882	136,088,794	62.3
Black—non-Hispanic	26,329	23,750,926	10.9
Hispanic	32,163	36,752,877	16.8
Other race—non-Hispanic	18,265	14,988,480	6.9
Multiracial—non-Hispanic	7473	2,908,153	1.3
Don’t know/Not sure/Refused/Missing	6926	3,977,914	1.8
Education			
Did not graduate high school	24,985	26,571,994	12.2
Graduated high school	96,704	59,410,029	27.2
Attended college or technical school	104,479	68,136,793	31.2
Graduated from college or technical school	143,766	63,609,214	29.1
Missing	1104	739,114	0.3
Income			
<$15,000	28,402	17,915,525	8.2
$15,000 to <$25,000	47,773	28,181,505	12.9
$25,000 to <$35,000	31,343	17,797,175	8.1
$35,000 to <$50,000	42,871	23,436,584	10.7
≥$50,000	160,301	96,433,310	44.1
Don’t know/Not sure/Missing	60,348	34,703,045	15.9

Other race—non-Hispanic includes American Indian or Alaskan Native only, non-Hispanic; Asian only, non-Hispanic; Native Hawaiian or other Pacific Islander only, non-Hispanic; and Other race only, non-Hispanic.

The mean consumption frequency of juice was 0.30 (SE = 0.002) times per day, and the mean consumption frequency of whole fruit was 1.06 (SE = 0.004) times per day. For CVD risk factors, 151,983 of 363,414 participants reported high blood pressure, and 130,399 of 346,233 participants reported high cholesterol. For CVD measures, 21,603 of 369,095 participants reported myocardial infarction (i.e., heart attack), 21,151 participants of 367,684 participants reported angina or CHD, and 16,489 of 370,024 reported stroke. The percentage of participants with and without each CVD risk factor and measure are shown in Table 3.

**TABLE 3:** Percentage of participants by cardiovascular disease (CVD) risk factor or measure (Yes or No)

CVD risk factor or measure	Group 1(Yes) (%)	Group 2(No) (%)
CVD risk factors		
High blood pressure (BPHIGH4)(*n* = 363,414)	33.7	66.3
High cholesterol (TOLDHI2)(*n* = 346,233)	31.9	68.1
CVD measures		
Heart attack (CVDINFR4)(*n* = 369,095)	4.3	95.7
Angina or coronary heart disease(CVDCRHD4) (*n* = 367,684)	4.0	96.0
Stroke (CVDSTRK3) (*n* = 370,024)	3.4	96.6

The following survey responses were not subpopulations of interest in the analysis for variables BPHIGH4, TOLDHI2, CVDINFR4, CVDCRHD4, and CVDSTRK3: Don’t Know/Not Sure (all variables), Refused (all variables), Not Asked or Missing (all variables), Yes, but female told only during pregnancy (BPHIGH4 only), Told borderline high or prehypertensive (BPHIGH4 only). Additionally, note that Yes and No groups are based on self-response and self-report of diagnosis of disease or risk factors. Frequencies were weighted to provide population-based estimates.

Fruit juice consumption frequency was higher in adults who self-reported high blood pressure ($\hat{\beta }$ = 0.0250, SE$\left(\hat{\beta }\right)$ = 0.0050, *P <* 0.0001), heart attack ($\hat{\beta }$ = 0.0753, SE$\left(\hat{\beta }\right)$ = 0.0148, *P <* 0.0001), angina or CHD ($\hat{\beta }$ = 0.0278, SE$\left(\hat{\beta }\right)$ = 0.0095, *P* = 0.0166), and stroke ($\hat{\beta }$ = 0.0719, SE$\left(\hat{\beta }\right)$ = 0.0115, *P <* 0.0001) when compared with the groups without the respective condition or risk factor. The high cholesterol variable differed: mean juice consumption frequency was lower in the group with high cholesterol compared with the group without high cholesterol ($\hat{\beta }$ = −0.01537, SE ($\hat{\beta }$) = 0.0053, *P* = 0.0174). Table 4 shows the group means, SEs, mean differences between groups, and *P* values for all independent samples *t* tests for juice consumption frequency.

**TABLE 4:** Independent samples *t* tests for daily consumption frequency of 100% fruit juice

Cardiovascular disease risk factor or measure	Group 1 (Yes)	Group 2 (No)	Comparison between groups	
	Mean ± SE	Mean ± SE	Difference	*P* value
CVD risk factors				
High blood pressure (BPHIGH4)	0.32 ± a	0.29 ± a	0.03	<0.0001
High cholesterol (TOLDHI2)	0.29 ± a	0.31 ± a	−0.012	0.0174
CVD measures				
Heart attack (CVDINFR4)	0.38 ± 0.01	0.30 ± a	0.^a^08	<0.0001
Angina or coronary heart disease (CVDCRHD4)	0.33 ± 0.01	0.30 ± a	0.03	0.0166
Stroke (CVDSTRK3)	0.37 ± 0.01	0.30 ± a	0.07	<0.0001

Abbreviation: CVD, cardiovascular disease.

The following survey responses were not subpopulations of interest in the analysis for variables BPHIGH4, TOLDHI2, CVDINFR4, CVDCRHD4, and CVDSTRK3: Don’t Know/Not Sure (all variables), Refused (all variables), Not Asked or Missing (all variables), Yes, but female told only during pregnancy (BPHIGH4 only), Told borderline high or pre-hypertensive (BPHIGH4 only). Additionally, note that Yes and No groups are based on self-response and self-report of diagnosis of disease or risk factors. *P* values were adjusted for the 5 CVD variables with Bonferroni corrections. A significance level of α = 0.05 was used. *t* tests were weighted to provide population-based estimates.

a Value is < 0.005 but >0.

Whole fruit consumption frequency was lower in adults who self-reported high blood pressure ($\hat{\beta }$ = −0.0763, SE$\left(\hat{\beta }\right)$ = 0.0075, *P <* 0.0001), high cholesterol ($\hat{\beta }$ = −0.0617, SE($\hat{\beta }$) = 0.0076, *P <* 0.0001), heart attack ($\hat{\beta }$ = −0.0862, SE($\hat{\beta }$) = 0.0136, *P <* 0.0001), CHD ($\hat{\beta }$ = −0.0547, SE($\hat{\beta }$) = 0.0155, *P* = 0.0020), and stroke ($\hat{\beta }$ = −0.0725, SE($\hat{\beta }$) = 0.0167, *P* < 0.0001) when compared with the groups without the respective condition/risk factor. Table 5 shows the group means, SEs, mean differences between groups, and *P* values for all independent samples *t* tests for whole fruit consumption frequency.

**TABLE 5:** Independent samples *t* tests for daily consumption frequency of whole fruit

Cardiovascular disease risk factor or measure	Group 1 (Yes)	Group 2 (No)	Comparison between groups	
	Mean ± SE	Mean ± SE	Difference	*P* value
CVD risk factors				
High blood pressure (BPHIGH4)	1.01 ± 0.01	1.09 ± a	−0.08	<0.0001
High cholesterol (TOLDHI2)	1.03 ± 0.01	1.09 ± a	−0.06	<0.0001
CVD measures				
Heart attack (CVDINFR4)	0.98 ± 0.01	1.07 ± a	−0.09	<0.0001
Angina or coronary heart disease (CVDCRHD4)	1.01 ± 0.02	1.07 ± a	−0.05	0.0020
Stroke (CVDSTRK3)	0.99 ± 0.02	1.07 ± a	−0.07	<0.0001

Abbreviation: CVD, cardiovascular disease.

The following survey responses were not subpopulations of interest in the analysis for variables BPHIGH4, TOLDHI2, CVDINFR4, CVDCRHD4, and CVDSTRK3: Don’t Know/Not Sure (all variables), Refused (all variables), Not Asked or Missing (all variables), Yes, but female told only during pregnancy (BPHIGH4 only), Told borderline high or prehypertensive (BPHIGH4 only). Additionally, note that Yes and No groups are based on self-response and self-report of diagnosis of disease or risk factors. *P* values were adjusted for the 5 CVD variables with Bonferroni corrections. A significance level of α = 0.05 was used. *t* tests were weighted to provide population-based estimates.

a Value is < 0.005 but >0.

Table 6 shows the estimates, SEs, estimated mean differences between groups, and adjusted probabilities from a regression model that includes independent variables of CVD (blood pressure, blood cholesterol, heart attack, CHD, and stroke) as well as age group, race/ethnic group, education and income levels, and smoking status with the dependent variable of 100% fruit juice intake frequency. Participants who self-reported high blood cholesterol had lower juice intake frequency, and participants who self-reported heart attack had higher juice intake frequency (blood cholesterol: $\hat{\beta }$ = −0.02345; SE ($\hat{\beta }$) = 0.0054; *P <* 0.0001; heart attack: $\hat{\beta }$ = 0.0430; SE ($\hat{\beta }$) = 0.0148; *P =* 0.0187). The participants who reported having experienced high blood pressure, CHD, or stroke did not significantly differ in fruit juice consumption frequency when accounting for age group, education group, income group, and smoking status (blood pressure: $\hat{\beta }$ = 0.0023; SE($\hat{\beta }$) = 0.0057; *P =* 1; CHD: $\hat{\beta }$ = 0.0048; SE($\hat{\beta }$) = 0.0098; *P =* 1; stroke: $\hat{\beta }$ = 0.0287; SE($\hat{\beta }$) = 0.0117; *P* = 0.0700).

**TABLE 6:** Regression analyses for impact of 100% fruit juice intake frequency on cardiovascular disease risk factors

CVD risk factor or measure	Group 1 (Yes)	Group 2 (No)	Comparison between groups	
	Estimate ± SE	Estimate ± SE	Difference	*P* value
CVD risk factors				
High blood pressure (BPHIGH4)	0.38 ± 0.02	0.38 ± 0.02	a	1
High cholesterol (TOLDHI2)	0.37 ± 0.02	0.40 ± 0.02	−0.02	<0.0001
CVD measures				
Heart attack (CVDINFR4)	0.42 ± 0.02	0.38± 0.02	0.04	0.0187
Angina or coronary heart disease (CVDCRHD4)	0.39 ± 0.02	0.38 ± 0.02	a	1
Stroke (CVDSTRK3)	0.41 ± 0.02	0.38 ± 0.02	0.03	0.0700

Abbreviation: CVD, cardiovascular disease.

The following survey responses were not subpopulations of interest in the analysis for variables BPHIGH4, TOLDHI2, CVDINFR4, CVDCRHD4, and CVDSTRK3: Don’t Know/Not Sure (all variables), Refused (all variables), Not Asked or Missing (all variables), Yes, but female told only during pregnancy (BPHIGH4 only), Told borderline high or pre-hypertensive (BPHIGH4 only). Additionally, note that Yes and No groups are based on self-response and self-report of diagnosis of disease or risk factors. *P* values were adjusted for the 5 CVD variables with Bonferroni corrections. A significance level of α = 0.05 was used. Regression models were adjusted for age group, education group, income group, and smoking status and were weighted to provide population-based estimates.

a Value is < 0.005 but >0.

Frequency of whole fruit consumption was associated with self-reported high blood pressure, blood cholesterol, heart attack, and CHD when accounting for age, education, income, and smoking status (blood pressure: $\hat{\beta }$ = −0.0869, SE($\hat{\beta }$) = 0.0083, *P <* 0.0001; blood cholesterol: $\hat{\beta }$ = −0.0812, SE($\hat{\beta }$) = 0.0080, *P <* 0.0001; heart attack: $\hat{\beta }$ = −0.0622; SE($\hat{\beta }$) = 0.0139, *P <* 0.0001; CHD: $\hat{\beta }$ = −0.0531; SE($\hat{\beta }$) = 0.0156, *P* = 0.0034). Self-reported stroke prevalence did not significantly differ by whole fruit consumption frequency when age, education, income, and smoking status were considered (stroke: $\hat{\beta }$ = −0.0374, SE ($\hat{\beta }$) = 0.0169, *P* = 0.1343). Table 7 shows the estimates, SEs, estimated mean differences between groups, and adjusted probabilities from a regression model that includes independent variables of CVD (blood pressure, blood cholesterol, heart attack, CHD, and stroke) as well as age group, race/ethnic group, education and income levels, and smoking status with the dependent variable of whole fruit intake frequency.

**TABLE 7:** Regression analyses for impact of whole fruit intake frequency on cardiovascular disease risk factors

CVD risk factor or measure	Group 1 (Yes)	Group 2 (No)	Comparison between groups	
	Estimate ± SE	Estimate ± SE	Difference	*P* value
CVD risk factors				
High blood pressure (BPHIGH4)	0.96 ± 0.02	1.04 ± 0.02	−0.09	<0.0001
High cholesterol (TOLDHI2)	0.97 ± 0.02	1.05 ± 0.02	−0.08	<0.0001
CVD Measures				
Heart attack (CVDINFR4)	0.96 ± 0.02	1.02 ± 0.02	−0.06	<0.0001
Angina or coronary heart disease (CVDCRHD4)	0.97 ± 0.03	1.02 ± 0.02	−0.05	0.0034
Stroke (CVDSTRK3)	0.98 ± 0.03	1.02 ± 0.02	−0.04	0.1343

Abbreviation: CVD, cardiovascular disease.

The following survey responses were not subpopulations of interest in the analysis for variables BPHIGH4, TOLDHI2, CVDINFR4, CVDCRHD4, and CVDSTRK3: Don’t Know/Not Sure (all variables), Refused (all variables), Not Asked or Missing (all variables), Yes, but female told only during pregnancy (BPHIGH4 only), Told borderline high or prehypertensive (BPHIGH4 only). Additionally, note that Yes and No groups are based on self-response and self-report of diagnosis of disease or risk factors. *P* values were adjusted for the 5 CVD variables with Bonferroni corrections. A significance level of α = 0.05 was used. Regression models were adjusted for age group, education group, income group, and smoking status and were weighted to provide population-based estimates.

## Discussion

Whole fruit consumption frequency was lower in groups with certain CVD risk factors (high blood pressure and blood cholesterol) as well as among adults living with CVD and CHD both before and after controlling for age, income, education, and smoking status. Whole fruit intake frequency was different among adults who self-reported stroke before, but not after, controlling for demographic and lifestyle covariates (age, income, education, and smoking status), indicating a potential role of these factors in the relationship between stroke and fruit consumption. These results could indicate the presence of a “sentinel event effect,” wherein individuals are motivated to change health behaviors, such as consuming fruits and vegetables, after a health scare such as a stroke [27]. Previous research and recommendations support consuming fruit for heart health. The American Heart Association recommends consuming a variety of fruits and vegetables—and limiting juice intake—to support heart health [28]. The Global Burden of Disease study attributed 13.5% of the risk for death from CVD and 14.9% of the risk for decreased disability-adjusted life years to low fruit intake, a leading dietary risk factor [29]. Finally, the DGA recommends consuming whole fruit as part of a healthy dietary pattern [2]. The difference in association before and after controlling for demographics and lifestyle factors could further indicate that some subpopulations are more likely to change dietary habits after a sentinel health event than others.

Only 2 CVD outcomes (high blood cholesterol and heart attack) were significantly associated with frequency of juice intake. The BRFSS dataset does not provide enough detailed information on juice intake frequency to further characterize a relationship between juice intake frequency and CVD risk. The BRFSS dataset also does not indicate the amount of juice consumed at each intake occasion. Previous systematic reviews and meta-analyses indicate a potential cardiovascular benefit to juice with intakes ≤200 mL [25,30,31]. The few detrimental associations with juice intake from our study could indicate groups of participants consuming greater amounts of juice.

Because of our unconventional approach to this research question (investigating how fruit intake frequency differs between adults with and without CVD or CVD indicators rather than how fruit or juice intake frequency may affect CVD risk factors), our results do not neatly align within the body of similar work on this topic. Although our results do not conflict with the body of previous research indicating a benefit of whole fruit consumption for CVD prevention [1,3,21,22,32], this analysis was also not structured to further add to that evidence base. In our study, individuals with high blood pressure and high cholesterol had lower whole fruit consumption frequency than the groups without high blood pressure and high cholesterol. As high blood pressure and high cholesterol are leading risk factors for CVD [33,34] and whole fruit consumption is associated with reduced likelihood of these risk factors [20], our findings also align with previous research that individuals who had experienced heart attack, angina or CHD, and stroke had lower fruit consumption frequency [[35], [36], [37]].

Both juice intake frequency and whole fruit consumption frequency differed among adults with and without high blood cholesterol and adults who did and did not report heart attacks, both before and after age, education, income, and smoking status were considered. The group with high cholesterol was an exception to the hypothesis; this group had a lower mean juice consumption frequency than others. Results from meta-analyses indicate either no effect of juice intake on blood cholesterol [25,38] or an impact only on total cholesterol [25], so the findings from our study may be due to confounding or the unique directionality of our approach. United States adults diagnosed with high cholesterol may be more likely than other groups to change their dietary intake before a more drastic health event occurs.

In addition, an important limitation of the self-reported high cholesterol data is that the 2019 BRFSS cholesterol awareness survey question (shown in Table 1) does not distinguish between LDL cholesterol and HDL cholesterol or total cholesterol and only asks survey participants if they have been told their “blood cholesterol is high” [26]. The lack of clarity between the cholesterol types, LDL cholesterol, which is associated with increased risk of CVD, and HDL cholesterol, which is associated with decreased risk of CVD, could have created confusion with participants and is a limitation of these data.

Only high cholesterol and heart attack prevalence were associated with greater frequency of juice consumption after controlling for covariates. Notably, the mean consumption frequency of juice was only 0.30 (±0.002) times per day with high variability in the data, and a moderate to large effect on disease risk with such infrequent intake may not be practical. Juice may not offer the same cardioprotective benefits as whole fruit [39]; however, additional research using longitudinal data and focusing on the cardioprotective nature of fruit compared with juice is needed for confirmation.

The results of this study suggest that adults with several adverse cardiovascular indicators have a lower fruit consumption frequency, even after accounting for age, education, income, and smoking status. One potential explanation for these results is that dietary fiber could be responsible for the CVD benefits associated with whole fruit consumption. As dietary fiber is primarily removed during the juicing process [6] and the consumption of dietary fiber is well established to reduce CVD risk [2,4], it offers one potential explanation for the differences between whole fruit and juice groups. Dietary fiber as a carrier of antioxidants, such as nonextractable polyphenols [15], offers another potential difference between whole fruit and juice. Relative bioaccessibility of polyphenols and carotenoids varies between fruit and juice because of their different matrices and nutrient profiles [40]. In vitro research indicates that the fiber present in whole fruit can decrease carotenoid bioaccessibility whereas juice, which lacks a fibrous matrix, serves as a source of more bioaccessible carotenoids [41]. These findings have been confirmed in human participants [42]. Yet, although carotenoids and extractable polyphenols in juice, such as hesperidin in orange juice, may be more bioaccessible than those in whole fruit, many studies do not measure nonextractable phenolic compounds [19,41,43,44]. The polyphenols contained in fruit and juice may also exert different effects in healthy individuals and in individuals with CVD [40].

In addition, processing methods used to prepare commercial juices can both increase and decrease polyphenol content [40,45]. For instance, commercial orange juicing processes release polyphenols from the seeds and peel, portions of the fruit that would otherwise not be consumed, into juice [40]. Heat treating, clarifying, and storing juice can decrease its antioxidant capacity and polyphenol content [7]. Even though the bioaccessibility of extractable phenolics was similar, freshly squeezed and pasteurized orange juice had 8-fold lower flavonoid content than fresh orange segments [41]. This study did not directly measure dietary fiber consumption or polyphenol content; the dietary fiber differences between whole fruit and juice are based on existing knowledge that most fiber is removed during the fruit juicing process [6].

### Limitations

This study has several limitations. Observational cross-sectional research is limited to provide data from a given timepoint and cannot be used to determine causation. BRFSS specifically collects data on consumption frequency rather than intake, so it is also not possible to stratify participants by low, moderate, and high intake of either whole fruit or juice consumption. Prediction equations can be applied to BRFSS data to estimate intake [5], and previous research [46] has indicated that these estimations align with data from NHANES; however, these equations were not applied in this study.

Participants may have changed their lifestyle after being diagnosed with CVD or a CVD risk factor, which could affect the analysis of the dependent variables related to the consumption of whole fruit and juice. Additionally, the BRFSS uses dietary recall information that depends on accurate recollection of participants. The survey questions instruct participants to only record intake frequency for 100% fruit juice and not to include “fruit-flavored drinks with added sugar or fruit juice you made at home and added sugar to” [47]. Yet, survey participants may misclassify sugar-sweetened beverages as juice or may have different perceptions of what constitutes whole fruit or juice. The 2019 BRFSS participants are not representative of the United States population. In addition, although this study controlled for some sociodemographic characteristics (age, smoking status, education, and income) that can affect CVD risk factors, other lifestyle factors such as physical activity, BMI, consumption of whole grains and vegetables, and consumption of sugar-sweetened beverages were not controlled in analyses. Not controlling for these factors may have confounded study results. In addition, this study did not evaluate comorbidities or potential overlap of participants consuming both fruit and juice, so it is possible that some of these diagnoses and frequencies overlap, which prevents us from having more precise conclusions. Finally, this study did not assess whether juice or whole fruit intake could predict CVD risk factors or conditions. CVD conditions and risk factors were treated as independent variables; however, juice intake frequency and whole fruit intake frequency could also be the independent variables, which may lead to different results.

## Conclusion

Total fruit intake in the United States adult population is substantially below federal recommendations with or without the inclusion of juice. Adults with high blood pressure, high cholesterol, heart attack, and CHD reported lower whole fruit consumption frequency than adults without these conditions, even after accounting for age, education, income, and smoking status. Differences in juice intake frequency persisted only for high cholesterol and heart attack after adjustment for covariates. Further research is warranted to examine how fruit intake differs among adults with and without CVD and to inform future public health recommendations.

## Author contributions

The authors’ responsibilities were as follows – BJK: designed and conducted the study and wrote the first draft; DGP, BJK: analyzed the data; AB, JMH, DGP, DT: provided support with these processes and edited the paper; and all authors: edited, read, and approved the final manuscript.

## Declaration of generative AI and AI-assisted technologies in the writing process

The authors declare that no generative AI or AI-assisted technologies were used in the writing of this manuscript.

## Data availability

All 2019 BRFSS data used in our analysis are publicly and freely available at: https://www.cdc.gov/brfss/annual_data/annual_2019.html.

## Funding

USDA Agricultural Research Service project grant #3062-51000-057-00D (JMH). BJK completed this research as a student in the Master of Science program in Nutrition at the University of North Dakota. Brendan is an employee with VDF FutureCeuticals, Inc., a part of the Van Drunen Farms Family of Companies and has received financial reimbursement for tuition-related expenses.

## Conflict of interest

BJK reports financial support was provided by VDF FutureCeuticals Inc. BJK reports a relationship with VDF FutureCeuticals Inc that includes: employment. The other authors declare that they have no known competing financial interests or personal relationships that could have appeared to influence the work reported in this paper.

## References

[1] USDA/HHS (2020).

[2] U.S. Department of Agriculture and U.S. Department of Health and Human Services (2025).

[3] 2025 Dietary Guidelines Advisory Committee (2024).

[4] Dietary Guidelines Advisory Committee (2020).

[5] Lee S.H. (2022). Adults meeting fruit and vegetable intake recommendations—United States, 2019. MMWR Morb. Mortal. Wkly. Rep..

[6] Ruxton C.H.S., Myers M. (2021). Fruit juices: are they helpful or harmful? An evidence review. Nutrients.

[7] Mavadiya H.B., Roh D., Ly A., Lu Y. (2025). Whole fruits versus 100% fruit juice: revisiting the evidence and its implications for US healthy dietary recommendations. Nutr. Bull..

[8] World Health Organization (2026). https://www.who.int/news-room/fact-sheets/detail/healthy-diet.

[9] Scheffers F.R., Boer J.M.A., Verschuren W.M.M., Verheus M., van der Schouw Y.T., Sluijs I. (2019). Pure fruit juice and fruit consumption and the risk of CVD: the European Prospective Investigation into Cancer and Nutrition-Netherlands (EPIC-NL) study. Br. J. Nutr..

[10] Federal Data Analysis Team and 2025 Dietary Guidelines Advisory Committee (2024).

[11] U.S. Department of Health and Human Services and U.S. Department of Agriculture (2005).

[12] USDA, HHS (2010).

[13] HHS, USDA (2015).

[14] U.S. Department of Agriculture Agricultural Research Service Nutrient Data Laboratory (2018).

[15] Saura-Calixto F., Saura-Calixto F., Pérez-Jiménez J. (2018). Non-extractable polyphenols and carotenoids: importance in human nutrition and health.

[16] Arranz S., Diaz M.E., Saura-Calixto F., Pérez-Jiménez J. (2018). Non-extractable polyphenols and carotenoids: importance in human nutrition and health.

[17] Arranz S., Saura-Calixto F., Shaha S., Kroon P.A. (2009). High contents of nonextractable polyphenols in fruits suggest that polyphenol contents of plant foods have been underestimated. J. Agric. Food Chem..

[18] Macagnan F.T., Bender A.B.B., Speroni C.S., Saura-Calixto F., Pérez-Jiménez J. (2018). Non-extractable polyphenols and carotenoids: importance in human nutrition and health.

[19] Pérez-Jiménez J., Díaz-Rubio M.E., Saura-Calixto F. (2013). Non-extractable polyphenols, a major dietary antioxidant: occurrence, metabolic fate and health effects. Nutr. Res. Rev.

[20] Dreher M.L. (2018). Whole fruits and fruit fiber emerging health effects. Nutrients.

[21] Slavin J.L., Lloyd B. (2012). Health benefits of fruits and vegetables. Adv. Nutr..

[22] Wallace T.C., Bailey R.L., Blumberg J.B., Burton-Freeman B., Chen C.O., Crowe-White K.M. (2020). Fruits, vegetables, and health: a comprehensive narrative, umbrella review of the science and recommendations for enhanced public policy to improve intake. Crit. Rev. Food. Sci. Nutr..

[23] Naghavi M., Ong K.L., Aali A., Ababneh H.S., Abate Y.H., Abbafati C. (2024). Global burden of 288 causes of death and life expectancy decomposition in 204 countries and territories and 811 subnational locations, 1990–2021: a systematic analysis for the Global Burden of Disease Study 2021. Lancet.

[24] Lichtenstein A.H., Khera A., Anderson C.A.M., Appel L.J., DeSilva D.M., Gardner C. (2026). 2026 Dietary Guidance to Improve Cardiovascular Health: a scientific statement from the American Heart Association. Circulation.

[25] D'Elia L., Dinu M., Sofi F., Volpe M., Strazzullo P. (2021). 100% Fruit juice intake and cardiovascular risk: a systematic review and meta-analysis of prospective and randomised controlled studies. Eur. J. Nutr..

[26] Centers for Disease Control and Prevention (2019).

[27] Boudreaux E.D., Bock B., O’Hea E. (2012). When an event sparks behavior change: an introduction to the sentinel event method of dynamic model building and its application to emergency medicine. Acad. Emerg. Med..

[28] Association A.H. (2023). https://www.heart.org/en/healthy-living/healthy-eating/add-color/how-to-eat-more-fruits-and-vegetables.

[29] Dong C., Bu X., Liu J., Wei L., Ma A., Wang T. (2022). Cardiovascular disease burden attributable to dietary risk factors from 1990 to 2019: a systematic analysis of the Global Burden of Disease study. Nutr. Metab. Cardiovasc. Dis..

[30] Liu Q., Chiavaroli L., Ayoub-Charette S., Ahmed A., Khan T.A., Au-Yeung F. (2023). Fructose-containing food sources and blood pressure: a systematic review and meta-analysis of controlled feeding trials. PLoS One.

[31] Liu Q., Ayoub-Charette S., Khan T.A., Au-Yeung F., Blanco Mejia S., de Souza R.J. (2019). Important food sources of fructose-containing sugars and incident hypertension: a systematic review and dose-response meta-analysis of prospective cohort studies. J. Am. Heart Assoc..

[32] USDA/HHS, Scientific report of the 2020 Dietary Guidelines for Americans..

[33] U.S. Centers for Disease Control and Prevention (CDC), Heart Disease Facts 2024 [Internet]. Available from: https://www.cdc.gov/heart-disease/data-research/facts-stats/index.html. Last updated: 10/24/24; Cited: 4/7/26.

[34] Palaniappan L.P., Allen N.B., Almarzooq Z.I., Anderson C.A.M., Arora P., Avery C.L. (2026). 2026 Heart Disease and Stroke Statistics: a report of US and global data from the American Heart Association. Circulation.

[35] Bazzano L.A., Serdula M.K., Liu S. (2003). Dietary intake of fruits and vegetables and risk of cardiovascular disease. Curr. Atheroscler. Rep..

[36] Oude Griep L.M., Monique Verschuren W.M., Kromhout D., Ocké M.C., Geleijnse J.M. (2011). Colours of fruit and vegetables and 10-year incidence of CHD. Br. J. Nutr..

[37] Zhang B., Pu L., Zhao T., Wang L., Shu C., Xu S. (2023). Global burden of cardiovascular disease from 1990 to 2019 attributable to dietary factors. J. Nutr..

[38] Liu K., Xing A., Chen K., Wang B., Zhou R., Chen S. (2013). Effect of fruit juice on cholesterol and blood pressure in adults: a meta-analysis of 19 randomized controlled trials. PLoS One.

[39] Sun T., Zhang Y., Ding L., Zhang Y., Li T., Li Q. (2023). The relationship between major food sources of fructose and cardiovascular outcomes: a systematic review and dose-response meta-analysis of prospective studies. Adv. Nutr..

[40] Ho K., Ferruzzi M.G., Wightman J.D. (2020). Potential health benefits of (poly)phenols derived from fruit and 100% fruit juice. Nutr. Rev..

[41] Aschoff J.K., Kaufmann S., Kalkan O., Neidhart S., Carle R., Schweiggert R.M. (2015). In vitro bioaccessibility of carotenoids, flavonoids, and vitamin c from differently processed oranges and orange juices [*Citrus sinensis* (L.) Osbeck]. J. Agric. Food Chem..

[42] Aschoff J.K., Rolke C.L., Breusing N., Bosy-Westphal A., Högel J., Carle R. (2015). Bioavailability of β-cryptoxanthin is greater from pasteurized orange juice than from fresh oranges - a randomized cross-over study. Mol. Nutr. Food Res..

[43] Carboni Martins C., Rodrigues R.C., Domeneghini Mercali G., Rodrigues E. (2022). New insights into non-extractable phenolic compounds analysis. Food Res. Int..

[44] Zeng Y., Zhou W., Yu J., Zhao L., Wang K., Hu Z. (2023). By-products of fruit and vegetables: antioxidant properties of extractable and non-extractable phenolic compounds. Antioxidants.

[45] Pyo Y.H., Jin Y.J., Hwang J.Y. (2014). Comparison of the effects of blending and juicing on the phytochemicals contents and antioxidant capacity of typical Korean kernel fruit juices. Prev. Nutr. Food Sci..

[46] Moore L.V., Dodd K.W., Thompson F.E., Grimm K.A., Kim S.A., Scanlon K.S. (2015). Using Behavioral Risk Factor Surveillance System data to estimate the percentage of the population meeting US Department of Agriculture Food Patterns Fruit and Vegetable Intake recommendations. Am. J. Epidemiol..

[47] Centers for Disease Control and Prevention, Using the new BRFSS module 2020 [Internet]. Available from: https://www.cdc.gov/nutrition/data-statistics/using-the-new-BRFSS-modules.html. Last updated: 9/1/20; Cited: 6/17/26.

